# Maternal sleep duration and neonatal birth weight: the Japan Environment and Children’s Study

**DOI:** 10.1186/s12884-021-03670-3

**Published:** 2021-04-12

**Authors:** Tsuyoshi Murata, Hyo Kyozuka, Toma Fukuda, Shun Yasuda, Akiko Yamaguchi, Seiichi Morokuma, Akiko Sato, Yuka Ogata, Kosei Shinoki, Mitsuaki Hosoya, Seiji Yasumura, Koichi Hashimoto, Hidekazu Nishigori, Keiya Fujimori, The Japan Environment and Children’s Study (JECS) Group, Michihiro Kamijima, Shin Yamazaki, Yukihiro Ohya, Reiko Kishi, Nobuo Yaegashi, Koichi Hashimoto, Chisato Mori, Shuichi Ito, Zentaro Yamagata, Hidekuni Inadera, Takeo Nakayama, Hiroyasu Iso, Masayuki Shima, Youichi Kurozawa, Narufumi Suganuma, Koichi Kusuhara, Takahiko Katoh

**Affiliations:** 1 Fukushima Regional Center for the Japan Environment and Children’s Study, 1 Hikarigaoka, Fukushima, 960-1295 Japan; 2 Department of Obstetrics and Gynecology, Fukushima Medical University School of Medicine, 1 Hikarigaoka, Fukushima, 960-1295 Japan; 3 Research Center for Environmental and Developmental Medical Sciences, Kyushu University, 3-1-1 Maidashi, Higashi-ku, Fukuoka, 812-0054 Japan; 4 Department of Health Sciences, Graduate School of Medical Sciences, Kyushu University, 3-1-1 Maidashi, Higashi-ku, Fukuoka, 812-8582 Japan; 5 Department of Pediatrics, Fukushima Medical University School of Medicine, 1 Hikarigaoka, Fukushima, 960-1295 Japan; 6 Department of Public Health, Fukushima Medical University School of Medicine, 1 Hikarigaoka, Fukushima, 960-1295 Japan; 7 Fukushima Medical Center for Children and Women, Fukushima Medical University, 1 Hikarigaoka, Fukushima, 960-1295 Japan

**Keywords:** Maternal sleep duration, Neonatal birth weight, Low birth weight infant, Small for gestational age infant, Macrosomia, Gestational weight gain

## Abstract

**Background:**

The adequate maternal sleep duration required for favorable obstetric outcomes is unknown. We evaluated the association between maternal sleep duration and low birth weight infants, small for gestational age infants, and macrosomia.

**Methods:**

Participants enrolled in the Japan Environment and Children’s Study, a nationwide birth cohort study, with singleton pregnancies after 22 weeks, who gave birth between 2011 and 2014 were enrolled and categorized into five groups according to maternal sleep duration during pregnancy: < 6.0 h, 6.0–7.9 h, 8.0–8.9 h, 9.0–9.9 h, and 10.0–12.0 h. We evaluated the association between maternal sleep duration and the incidence of low birth weight infants (< 2500 g), very low birth weight infants (< 1500 g), small for gestational age infants, and macrosomia (> 4000 g), with women with maternal sleep duration of 6.0–7.9 h as the reference, using a multiple logistic regression model.

**Results:**

In total, 82,171 participants were analyzed. The adjusted odds ratios (95% confidence intervals) for low birth weight infants in women with maternal sleep duration of 9.0–9.9 h and 10.0–12.0 h and for small for gestational age infants in women with maternal sleep duration of 9.0–9.9 h were 0.90 (0.83–0.99), 0.86 (0.76–0.99), and 0.91 (0.82–0.99), respectively, before adjusting for excessive gestational weight gain. No significant association was observed between maternal sleep duration and these outcomes after adjusting for excessive gestational weight gain. Among women with appropriate gestational weight gain, the adjusted odds ratios (95% confidence intervals) for low birth weight infants and for small for gestational age infants with sleep duration of 9.0–9.9 h were 0.88 (0.80–0.97) and 0.87 (0.78–0.97), respectively.

**Conclusions:**

Maternal sleep duration of 9.0–9.9 h was significantly associated with the decreased incidence of low birth weight infants and small for gestational age infants in pregnant women with appropriate gestational weight gain, compared with that of 6.0–7.9 h. Care providers should provide proper counseling regarding the association between maternal sleep duration and neonatal birth weight and suggest comprehensive maternal lifestyle modifications to prevent low birth weight and small for gestational age infants.

**Supplementary Information:**

The online version contains supplementary material available at 10.1186/s12884-021-03670-3.

## Background

Neonatal birth weight, which is related to perinatal morbidity and mortality [1–4], is affected by several obstetric complications, including preterm births (PTB), fetal growth restriction (FGR), and preeclampsia [5, 6]. Specifically, infants with birth weight < 1500 g, defined as very low birth weight (VLBW) infants, are severely premature and may experience increased mortality [7]. Additionally, small for gestational age (SGA) infants are also at an increased risk of neonatal and post-neonatal mortality [4, 8, 9]. Further, low birth weight (LBW) and SGA infants are associated with an increased risk of coronary artery disease, diabetes mellitus, and arterial hypertension in adulthood, as described in the Baker hypothesis [10], which has been revised to the concept of developmental origins of health and disease [11]. Conversely, macrosomia, defined as birth weight > 4000 g, is also associated with a risk of morbidity in infants [2, 12].

Several modifiable factors, including maternal pre-pregnancy body weight, gestational weight gain (GWG), and diet, have a major impact on the neonatal birth weight [5, 12–14]. Similarly, maternal sleep duration (MSD) during pregnancy also affects obstetric outcomes [15–17]. However, the association between MSD and neonatal birth weight remains unclear [15, 18]. Previous studies have reported reduced MSD as a risk factor for the incidence of SGA infants [19, 20], but Morokuma et al. reported no such association in a study of 8631 participants [21]. Moreover, the appropriate MSD required to prevent LBW infants, SGA infants, and macrosomia has not been elucidated. As sleep is often disturbed in pregnant women [15], appropriate MSD could be a great concern.

The present study evaluated the association between MSD and LBW infants, VLBW infants, SGA infants, and macrosomia using the data from a nationwide Japanese birth cohort study.

## Methods

### Study design

In this study, we retrospectively analyzed the data from the Japan Environment and Children’s Study (JECS), which is a nationwide, government-funded, prospective birth cohort study that was started in January 2011 to investigate the effects of environmental factors on children’s health [22, 23]. Briefly, the JECS is funded directly by the Ministry of the Environment, Japan and involves collaboration among the Programme Office (National Institute for Environmental Studies), the Medical Support Centre (National Centre for Child Health and Development), and 15 Regional Centres (Hokkaido, Miyagi, Fukushima, Chiba, Kanagawa, Koshin, Toyama, Aichi, Kyoto, Osaka, Hyogo, Tottori, Kochi, Fukuoka, and South Kyushu/Okinawa) [23]. The eligibility criteria for expectant mothers to participate in the JECS were as follows: (1) residing in the study areas at the time of recruitment and expected to continually reside in Japan for the foreseeable future; (2) an expected delivery date between August 1, 2011 and mid-2014; and (3) capable of participating in the study without difficulty (i.e., able to comprehend the Japanese language and complete the self-administered questionnaires).

We applied either or both of the following recruitment protocols: (1) recruitment at the time of the first prenatal examination at the cooperating obstetric facilities; and (2) recruitment at local government offices issuing a pregnancy journal, called the Maternal and Child Health Handbook, that is given to all expecting mothers in Japan before they receive municipal services for pregnancy, delivery, and childcare. We contacted pregnant women through cooperating health care providers and/or local government offices issuing the Maternal and Child Health Handbooks and registered those willing to participate. Self-administered questionnaires, which were completed by the women during the first and second/third trimesters, were used to collect information on demographic factors, medical and obstetric history, physical and mental health, lifestyle, occupation, environmental exposure at home and at the workplace, housing conditions, and socioeconomic status [23].

In addition to the ethics committees of all participating institutions, the Ministry of the Environment’s Institutional Review Board on Epidemiological Studies reviewed and approved the JECS protocol (No. 100910001). The JECS was conducted in accordance with the principles of the Declaration of Helsinki and other national regulations and guidelines. Written informed consent was obtained from all participants.

### Data collection

The current analysis used the data set released in June 2016 (data set: jecs-ag-20160424). Specifically, we used three types of data: (1) M-T1, obtained from a self-reported questionnaire that was collected during the first trimester (the first questionnaire) and included questions regarding maternal medical background; (2) M-T2, obtained from a self-reported questionnaire that was collected during the second or third trimester (second questionnaire) and included questions regarding lifestyle and socioeconomic status; and (3) Dr-0m, collected from the medical record transcripts provided by each participant’s institution that included data for obstetrical outcomes such as gestational age, birth weight, neonatal sex, and maternal body weight.

The participants with singleton pregnancies after 22 weeks were included in the present study. Women with abortion, stillbirths, and missing information were excluded from the analysis. There were no significant differences in characteristics between those included in and excluded from the analysis (data not shown).

### Exposure variables

We calculated MSD using the data of the questionnaire in M-T2 [24]. The participants submitted data about their bedtime and waking up time and were categorized into five groups according to MSD during pregnancy: MSD < 6.0 h, MSD 6.0–7.9 h, MSD 8.0–8.9 h, MSD 9.0–9.9 h, and MSD 10.0–12.0 h. These categories were based on the report of the Ministry of Health, Labour and Welfare, Japan [25], which suggests 6.0–7.9 h of sleep as the appropriate sleep duration for general adults to prevent several health problems. Participants who reported MSD > 12.0 h were excluded from the present study because they were considered to have reported inaccurate MSDs.

### Obstetric outcomes and confounding factors

We classified LBW infants into two categories: LBW infants < 2500 g and VLBW infants < 1500 g [1]. SGA infants were defined as a birth weight < 1.5 standard deviations, corrected for parity, gestational age, and sex, according to the “New Japanese neonatal anthropometric charts for gestational age at birth” [8]. Macrosomia was defined as neonatal birth weight > 4000 g [12].

We considered the following parameters as potential confounding factors: maternal age, body mass index (BMI) before pregnancy, parity, maternal smoking status, maternal alcohol consumption status, maternal educational status, annual household income, PTB before 37 weeks, and GWG. Maternal age was categorized into three groups: < 20 years, 20–29 years, and ≥ 30 years, based on a previous study that showed that maternal age was related to certain obstetric outcomes, such as PTB, LBW infants, and SGA infants [26]. BMI before pregnancy was categorized into three groups: < 18.5 kg/m^2^, 18.5–24.9 kg/m^2^, and ≥ 25.0 kg/m^2^ [27, 28]. Parity was categorized into two groups: nulliparous and multiparous. We requested maternal participants to provide information about their smoking status by choosing one of the following: “Currently smoking,” “Never,” “Previously did but quit before realizing current pregnancy,” and “Previously did but quit after realizing current pregnancy.” The maternal participants who chose “Currently smoking” comprised the smoking category, whereas the other participants comprised the non-smoking category. We also asked the maternal participants to provide information about their alcohol consumption status by choosing one of the following: “Never drank,” “Quit drinking before pregnancy,” “Quit drinking during early pregnancy,” and “Kept drinking during pregnancy” [29]. The maternal participants who chose “Kept drinking during pregnancy” comprised the drinking category, whereas the other participants comprised the non-drinking category. Maternal educational status was categorized into four groups based on the number of years of education: junior high school, < 10 years; high school, 10–12 years; professional school or university, 13–16 years; and graduate school, ≥17 years. Annual household income was categorized into four groups: < 2,000,000 JPY, 2,000,000–5,999,999 JPY, 6,000,000–9,999,999 JPY, and ≥ 10,000,000 JPY. PTB was defined as birth before 37 weeks of gestation, the record of which was collected from Dr-0m data. The data for GWG were obtained from Dr-0m data, which included information about body weight before pregnancy (kg) and body weight just before delivery (kg). GWG was defined as body weight just before delivery minus body weight before pregnancy (kg). We defined appropriate GWG as < 12 kg and excessive GWG as ≥ 12 kg, according to the criteria aimed at appropriate birth weight, described by the Ministry of Health, Labour and Welfare, Japan [27, 28], where the appropriate GWG is defined as 9–12 kg for women with pre-pregnancy BMI below 18.8 kg/m^2^ and 7–12 kg for women with pre-pregnancy BMI between 18.5–24.9 kg/m^2^, and individually assigned for women with pre-pregnancy BMI over 25.0 kg/m^2^. These confounding factors were chosen on the basis of their clinical importance [5, 13, 14]. Since GWG in particular is indicative of lifestyle, it was thought to be a significant confounding factor.

### Statistical analyses

Participant characteristics were summarized according to the maternal sleeping status. One-way analysis of variance and the Kruskal-Wallis test were used to compare continuous variables among different MSD groups, according to the difference in the distribution of data. The chi-square test was used to compare categorical variables.

Initially, crude odds ratios (cORs), adjusted odds ratios (aORs), and 95% confidence intervals (CI) for LBW infants, VLBW infants, SGA infants, and macrosomia were calculated using a multiple logistic regression model, with women with MSD of 6.0–7.9 h as the reference. In Model 1, the odds ratios for LBW infants and VLBW infants were adjusted for maternal age, BMI before pregnancy, parity, maternal smoking status, maternal alcohol consumption status, maternal educational status, annual household income, and PTB before 37 weeks. The odds ratio for SGA infants was adjusted for maternal age, BMI before pregnancy, maternal smoking status, maternal alcohol consumption status, maternal educational status, and annual household income. The odds ratio for macrosomia was adjusted for maternal age, BMI before pregnancy, parity, maternal smoking status, maternal alcohol consumption status, maternal educational status, and annual household income. In Model 2, excessive GWG was added as a confounding factor (in addition to those of Model 1) to calculate the aORs for these outcomes.

Further, we stratified the participants based on GWG, and cORs and aORs for LBW infants and SGA infants were calculated using a multiple logistic regression model, with women with MSD of 6.0–7.9 h as the reference, using the same confounding factors as Model 1.

SPSS version 26 (IBM Corp., Armonk, NY) was used to perform the statistical analyses. Differences with *p*-values < 0.05 were considered statistically significant.

## Results

**Fig. 1:**
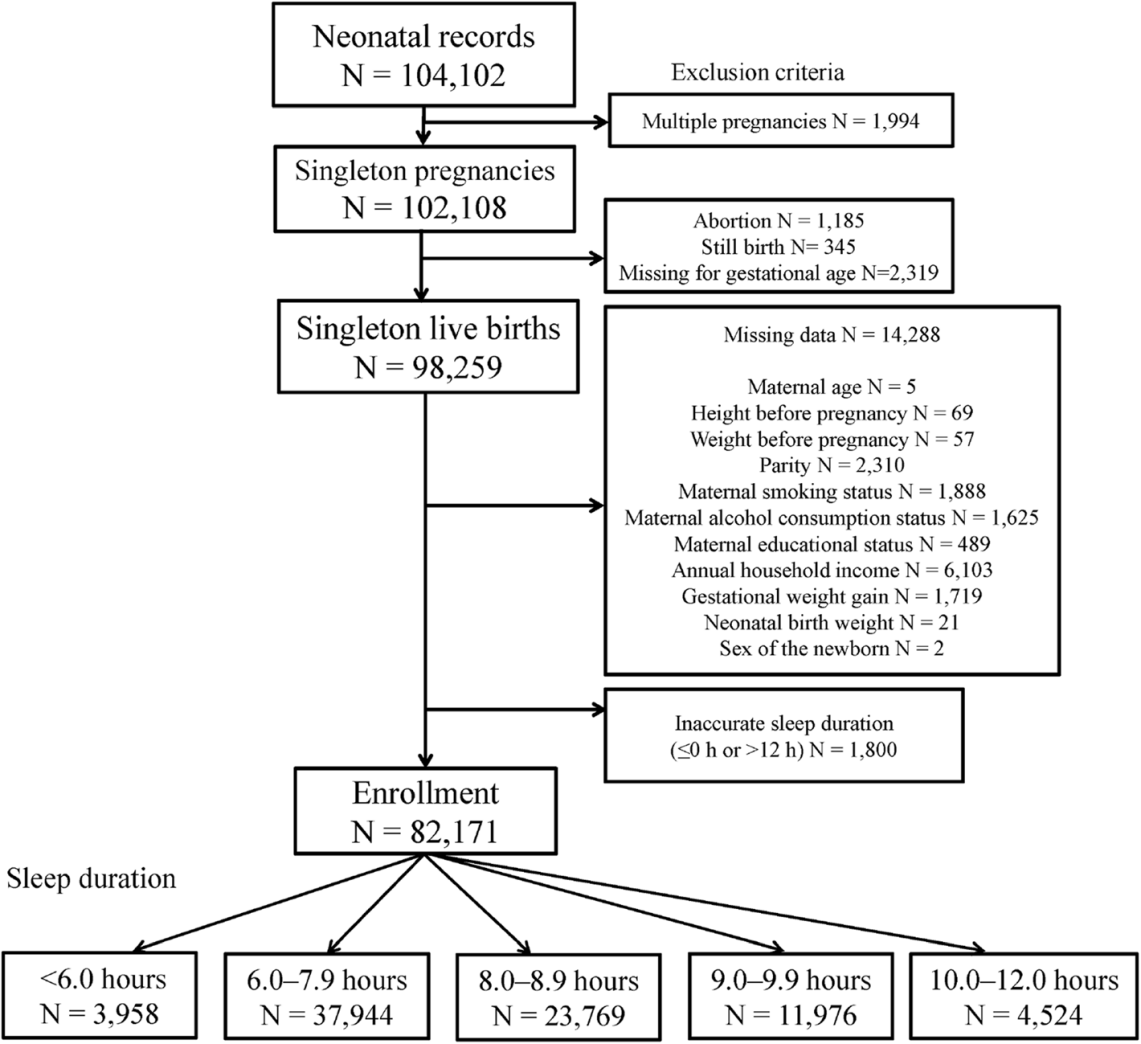
Flowchart depicting enrollment of the participants in the study

The total number of fetal records during 2011–2014 was 104,102. After applying our inclusion criteria, 82,171 participants were eligible for this study (Fig. 1). There were missing data regarding maternal age in 5 cases, height before pregnancy in 69, weight before pregnancy in 57, parity in 2310, maternal smoking status in 1888, maternal alcohol consumption status in 1625, maternal educational status in 489, annual household income in 6103, GWG in 1719, neonatal birth weight in 21, sex of the newborn in 2, and inaccurate MSD (≤0 or > 12 h) in 1800. Among the 82,171 participants, 3958 (4.8%) had MSD of < 6.0 h, 37,944 (46.2%) had MSD of 6.0–7.9 h, 23,769 (28.9%) had MSD of 8.0–8.9 h, 11,976 (14.6%) had MSD of 9.0–9.9 h, and 4524 (5.5%) had MSD of 10.0–12.0 h.

Table 1 summarizes the maternal background and obstetric outcomes based on the MSD groups. All maternal background characteristics were significantly affected by MSD. The incidence of LBW infants was significantly lower in women with MSD of 9.0–9.9 and 10.0–12.0 h than in the other groups. No significant differences were observed in the incidence of PTB, VLBW infants, SGA infants, and macrosomia among the groups.

**Table 1:** Maternal medical background and obstetric outcomes of participants according to maternal sleep duration status

	MSD < 6.0 h	MSD 6.0–7.9 h	MSD 8.0–8.9 h	MSD 9.0–9.9 h	MSD 10.0–12.0 h	p-value
Variable	*n* = 3958	*n* = 37,944	*n* = 23,769	*n* = 11,976	*n* = 4524	
Maternal medical background						
Maternal age (years), mean ± SD	31.7 ± 5.5	31.7 ± 4.9	31.5 ± 4.7	30.9 ± 4.8	29.1 ± 5.2	< 0.001
BMI (kg/m^2^) before pregnancy, mean ± SD	21.4 ± 3.6	21.2 ± 3.3	21.3 ± 3.2	21.3 ± 3.4	21.3 ± 3.4	< 0.001
Nulliparous, % (n)	51.6 (2043)	47.7 (18,096)	32.7 (7761)	25.0 (2992)	37.1 (1679)	< 0.001
Smoking during pregnancy, % (n)	8.9 (352)	4.6 (1751)	4.1 (971)	4.0 (475)	6.3 (284)	< 0.001
Alcohol consumption, % (n)	3.8 (149)	2.7 (1008)	3.0 (707)	3.2 (384)	2.5 (114)	< 0.001
Maternal educational status, % (n)						
< 10 years	7.3 (289)	3.8 (1442)	3.9 (929)	4.9 (588)	9.0 (406)	< 0.001
10–12 years	35.1 (1390)	29.8 (11,296)	30.0 (7135)	31.5 (3778)	37.6 (1699)	< 0.001
13–16 years	56.3 (2227)	64.8 (24,599)	64.6 (15,359)	62.2 (7445)	52.7 (2383)	< 0.001
≥ 17 years	1.3 (52)	1.6 (607)	1.5 (346)	1.4 (165)	0.8 (36)	< 0.001
Annual household income, % (n)						
< 2,000,000 JPY	7.6 (302)	4.5 (1708)	5.1 (1209)	7.2 (862)	11.8 (533)	< 0.001
2,000,000–5,999,999 JPY	66.3 (2624)	65.8 (24,986)	68.1 (16,178)	70.4 (8426)	73.7 (3333)	< 0.001
6,000,000–9,999,999 JPY	21.6 (856)	25.1 (9524)	22.5 (5354)	18.7 (2244)	12.0 (544)	< 0.001
≥ 10,000,000 JPY	4.4 (176)	4.5 (1726)	4.3 (1028)	3.7 (444)	2.5 (114)	< 0.001
GWG (kg), mean ± SD	10.3 ± 4.3	10.2 ± 4.8	10.3 ± 5.9	10.2 ± 4.1	10.6 ± 4.3	< 0.001
Excessive GWG, ≥ 12 kg, % (n)	33.6 (1331)	30.8 (11,690)	31.2 (7423)	31.9 (3819)	36.9 (1669)	< 0.001
Obstetric outcomes						
PTB, < 37 weeks, % (n)	4.7 (185)	4.4 (1660)	4.5 (1058)	4.3 (516)	4.5 (205)	0.86
Neonatal birth weight (g), mean ± SD	3017 ± 419	3022 ± 412	3037 ± 410	3044 ± 406	3030 ± 410	< 0.001
LBW infants < 2500 g, % (n)	8.3 (330)	8.2 (3101)	7.6 (1803)	7.0 (837)	7.2 (327)	< 0.001
VLBW infants < 1500 g, % (n)	0.5 (20)	0.5 (177)	0.4 (101)	0.4 (51)	0.6 (25)	0.87
SGA infants, % (n)	4.7 (187)	5.1 (1951)	4.9 (1173)	4.7 (558)	5.0 (224)	0.25
Macrosomia, > 4000 g, % (n)	1.0 (41)	0.8 (318)	0.9 (211)	1.0 (119)	0.8 (35)	0.38

*Abbreviations: MSD* maternal sleep duration, *SD* standard deviation, *BMI* body mass index, *GWG* gestational weight gain, *PTB* preterm births, *LBW* low birth weight, *VLBW* very low birth weight, *SGA* small for gestational age

Table 2 shows the cORs and aORs for LBW infants, VLBW infants, SGA infants, and macrosomia among all groups, with women with MSD of 6.0–7.9 h as a reference. The aORs (95% CIs) for LBW infants in women with MSD of 9.0–9.9 and 10.0–12.0 h and for SGA infants in women with MSD of 9.0–9.9 h were 0.90 (0.83–0.99), 0.86 (0.76–0.99), and 0.91 (0.82–0.99), respectively, before adjusting for excessive GWG in Model 1. However, no significant association was observed between MSD and these outcomes after adjusting for excessive GWG in Model 2. Additionally, there was no significant association between MSD and VLBW infants and macrosomia in Models 1 and 2.

**Table 2:** cORs, aORs, and 95% CIs of obstetric complications stratified by maternal sleep duration

Obstetric outcomes	LBW infants	VLBW infants	SGA infants	Macrosomia
	< 2500 g	< 1500 g		
cORs and aORs (95% CI)				
MSD < 6.0 h				
cORs	1.02 (0.91–1.15)	1.08 (0.68–1.72)	0.92 (0.78–1.07)	1.24 (0.89–1.72)
Model 1	0.95 (0.83–1.08)	1.01 (0.62–1.65)	0.88 (0.75–1.02)	1.23 (0.89–1.71)
Model 2	0.95 (0.83–1.09)	1.02 (0.62–1.67)	0.89 (0.76–1.03)	1.19 (0.86–1.66)
MSD 6.0–7.9 h				
	Ref	Ref	Ref	Ref
MSD 8.0–8.9 h				
cORs	0.92 (0.87–0.98)	0.91 (0.71–1.16)	0.96 (0.89–1.03)	1.06 (0.89–1.26)
Model 1	0.96 (0.90–1.03)	0.95 (0.73–1.23)	0.96 (0.90–1.04)	1.01 (0.84–1.20)
Model 2	0.97 (0.91–1.04)	0.95 (0.73–1.24)	0.97 (0.90–1.04)	0.99 (0.83–1.19)
MSD 9.0–9.9 h				
cORs	0.84 (0.78–0.91)	0.91 (0.67–1.25)	0.90 (0.82–0.99)	1.19 (0.96–1.47)
Model 1	0.90 (0.83–0.99)	0.99 (0.71–1.37)	0.91 (0.82–0.99)	1.10 (0.89–1.36)
Model 2	0.92 (0.84–1.00)	0.99 (0.71–1.38)	0.91 (0.83–1.00)	1.08 (0.87–1.34)
MSD 10.0–12.0 h				
cORs	0.88 (0.88–0.99)	1.19 (0.78–1.80)	0.96 (0.83–1.11)	0.92 (0.65–1.31)
Model 1	0.86 (0.76–0.99)	1.27 (0.81–1.99)	0.95 (0.83–1.10)	0.89 (0.63–1.27)
Model 2	0.90 (0.78–1.03)	1.31 (0.83–2.06)	0.98 (0.85–1.13)	0.86 (0.60–1.23)

*Abbreviations: LBW* low birth weight, *VLBW* very low birth weight, *SGA* small for gestational age, *MSD* maternal sleep duration, *Ref* reference, *cORs* crude odds ratios, *aORs* adjusted odds ratios, *95% CIs* 95% confidence intervals

Maternal age, body mass index before pregnancy, parity, maternal smoking status, maternal alcohol consumption status, maternal educational status, annual household income, and preterm birth before 37 weeks were used as confounding factors in Model 1. Excessive gestational weight gain was added as a confounding factor (in addition to those of Model 1) in Model 2

Table 3 shows the cORs and aORs for LBW infants and SGA infants among all groups, with women with MSD of 6.0–7.9 h as a reference, after stratification by GWG. Among women with appropriate GWG, the aORs (95% CIs) for LBW infants and SGA infants in women with MSD of 9.0–9.9 h were 0.88 (0.80–0.97) and 0.87 (0.78–0.97), respectively. However, no significant association was observed between MSD and these outcomes in women with excessive GWG.

**Table 3:** cORs, aORs, and 95% CIs of infant groups by women’s gestational weight gain

Obstetric outcome	Appropriate GWG		Excessive GWG	
	LBW infants	SGA infants	LBW infants	SGA infants
cORs and aORs (95% CI)				
MSD < 6.0 h				
cORs	1.07 (0.94–1.22)	0.97 (0.82–1.15)	0.92 (0.68–1.24)	0.79 (0.55–1.14)
aORs	0.98 (0.84–1.13)	0.92 (0.78–1.09)	0.85 (0.61–1.17)	0.75 (0.52–1.08)
MSD 6.0–7.9 h				
	Ref	Ref	Ref	Ref
MSD 8.0–8.9 h				
cORs	0.94 (0.88–1.00)	0.95 (0.88–1.03)	0.86 (0.73–1.00)	1.00 (0.84–1.18)
aORs	0.99 (0.92–1.06)	0.96 (0.88–1.04)	0.90 (0.76–1.06)	1.00 (0.84–1.19)
MSD 9.0–9.9 h				
cORs	0.83 (0.76–0.90)	0.87 (0.78–0.97)	0.97 (0.80–1.17)	1.08 (0.88–1.33)
aORs	0.88 (0.80–0.97)	0.87 (0.78–0.97)	1.10 (0.89–1.35)	1.07 (0.87–1.32)
MSD 10.0–12.0 h				
cORs	0.90 (0.79–1.03)	0.96 (0.81–1.13)	1.01 (0.78–1.31)	1.14 (0.85–1.51)
aORs	0.89 (0.76–1.04)	0.87 (0.76–1.01)	0.92 (0.69–1.23)	1.08 (0.81–1.44)

*Abbreviations: GWG* gestational weight gain, *LBW* low birth weight, *SGA* small for gestational age, *MSD* maternal sleep duration *Ref* reference, *cORs* crude odds ratios, *aORs* adjusted odds ratios, *95% CIs* 95% confidence intervals

Maternal age, body mass index before pregnancy, parity, maternal smoking status, maternal alcohol consumption status, maternal educational status, annual household income, and preterm birth before 37 weeks were used as the confounding factors

## Discussion

The present study showed an association between MSD over 9.0 h and the decreased incidence of LBW and SGA infants. However, this association was not sustained after adjusting for excessive GWG, which implies that both long MSD and excessive GWG are associated with a decreased incidence of LBW and SGA infants. Additionally, MSD of 9.0–9.9 h was significantly associated with the decreased incidence of LBW and SGA infants in women with appropriate GWG.

The present study, using a large birth cohort, showed that both MSD and GWG had an association with neonatal birth weight. Previous studies with relatively small sample sizes have shown a conflicting relationship between MSD and neonatal birth weight [15, 19–21]. Based on the findings of the present study, we speculate that long MSD, along with GWG, may be associated with the decreased incidence of LBW and SGA infants [14]. Although the effect of MSD on GWG is not clearly defined [30, 31], recent studies have reported that excessive sleep duration increases obesity in non-pregnant adults [32, 33]. The fetuses in mothers with excessive GWG may receive more nutrients and experience greater growth through increased plasma volume, which may increase cardiac output and utero-placental blood flow compared to those in mothers with insufficient GWG [34, 35]. Therefore, sufficient GWG by proper diet and sufficient MSD are required to prevent the incidence of LBW and SGA infants; however, the disadvantages of maternal obesity should be considered [36, 37].

The direct effects of MSD alone on obstetric outcomes in the appropriate GWG group have not been clarified yet. Maternal inflammatory stress has been reported to be related to several obstetric outcomes such as PTB, FGR, and preeclampsia [38–40]. Previous studies have also reported that disturbed maternal sleep may cause adverse obstetric outcomes, with augmentation of maternal inflammatory response [15, 41]. Increased inflammation may interfere with the remodeling of spiral arteries in the placenta, thereby leading to PTB, FGR, and preeclampsia [42–44]. Thus, preventing reduced MSD may reduce maternal inflammation and prevent these adverse obstetric outcomes. Further, maternal inflammatory stress is affected by lifestyle, including dietary habits and exercise [45, 46], and comprehensive lifestyle modification may help in reducing inflammatory stress.

However, MSD of 10.0–12.0 h was not associated with a decreased incidence of LBW and SGA infants in women with appropriate GWG. This may be because MSD of 10.0–12.0 h might be affected by maternal diseases, conditions, and behaviors, such as depression, excessive mental stress, and use of sleeping pills [24, 47, 48], which may potentially decrease neonatal birth weight [49–51]. Moreover, because excessively long MSD may have other unfavorable effects, including excessive GWG [32, 33], we do not suggest that pregnant women should have MSDs of over 10.0 h.

The strength of the present study is that the aORs for LBW and SGA infants provide clear information for perinatal counselling. Owing to the large study population of > 80,000 participants, our results should be considered reliable. Because pregnant women have more sleep problems, affected by gestational age and hormonal changes [24], than their non-pregnant counterparts, the association between MSD and fetal and neonatal health may be a great concern for pregnant women. There is no consensus on the appropriate MSD required to prevent adverse obstetric outcomes. Therefore, the present study would be helpful in suggesting the appropriate MSD required to prevent the incidence of LBW and SGA infants. Furthermore, because the JECS is a prospective cohort study, elucidation of long-term childhood outcomes based on MSD and neonatal birth weight in the future would strengthen the conclusions of this study.

The present study has some limitations. First, MSD in the present study was based on self-reported questionnaire data, which might have resulted in an inaccurate calculation of actual MSD. Nevertheless, several studies have shown a moderate correlation between self-reported and objectively-evaluated sleep duration measurements [52, 53]. Moreover, MSD is a volatile index because it varies daily in individuals and may vary with gestational age [24]. Careful interpretation is needed regarding these instabilities of MSD. Further study with polysomnography and unified gestational age may address this limitation. Second, we did not account for the quality of sleep by evaluating factors such as time zone, division, sleep location, and next to whom the participants are sleeping. Sleeping habits, including night and midday sleep durations, may also affect the infants’ weights. We evaluated the MSD as a simple quantitative measurement of maternal sleep to easily counsel pregnant women. Careful interpretation of the results is needed because the quality of maternal sleep, in addition to MSD, may also affect the obstetric outcomes. Finally, as this was a retrospective observational study, we could not clarify a cause-and-effect relationship. Although there was a significant association between MSD and the incidence of LBW and SGA infants in a certain setting, careful interpretation of the results is needed.

## Conclusions

This study revealed that both MSD over 9.0 h and excessive GWG were significantly associated with the decreased incidence of LBW and SGA infants, and that MSD of 9.0–9.9 h was significantly associated with the decreased incidence of LBW and SGA infants in women with appropriate GWG. It is important for care providers to provide the latest data regarding the association between MSD and neonatal birth weight for proper counselling, and to suggest comprehensive modifications in the lifestyles of pregnant women, including sufficient MSD, to prevent the incidence of LBW and SGA infants. The present study may shed some light on the appropriate MSD required to prevent the incidence of LBW and SGA infants.

## Supplementary Information

**Additional file 1.**

## Data Availability

Data are unsuitable for public deposition due to ethical restrictions and legal framework of Japan. It is prohibited by the Act on the Protection of Personal Information (Act No. 57 of 30 May 2003, amendment on 9 September 2015) to publicly deposit the data containing personal information. Ethical Guidelines for Epidemiological Research enforced by the Japan Ministry of Education, Culture, Sports, Science and Technology and the Ministry of Health, Labour and Welfare also restricts the open sharing of the epidemiologic data. All inquiries about access to data should be sent to: jecs-en@nies.go.jp. The person responsible for handling enquiries sent to this e-mail address is Dr. Shoji F. Nakayama, JECS Programme Office, National Institute for Environmental Studies.

## Abbreviations

aOR: Adjusted odds ratio
BMI: Body mass index
CI: Confidence interval
cOR: Crude odds ratio
FGR: Fetal growth restriction
GWG: Gestational weight gain
JECS: Japan Environment and Children’s Study
LBW: Low birth weight
MSD: Maternal sleep duration
PTB: Preterm births
SGA: Small for gestational age
VLBW: Very low birth weight
