# Supplementary material for: Maternal sleep duration and neonatal birth weight: the Japan Environment and Children’s Study
Source: BMC Pregnancy Childbirth. 2021 Apr 12;21:295. doi: 10.1186/s12884-021-03670-3 (PMC8042950; doi:10.1186/s12884-021-03670-3)
Supplement: Supplementary file 1 — Additional file 1. [file 12884_2021_3670_MOESM1_ESM.docx]

Ethics Committee of all participating institutions

1) National Center for Child Health and Development

2) National Institute for Environmental Studies

3) Hokkaido University

4) Sapporo Medical University

5) Asahikawa Medical University

6) Japanese Red Cross Hokkaido College of Nursing

7) Tohoku University

8) Fukushima Medical University

9) Chiba University

10) Yokohama City University

11) University of Yamanashi

12) Shinshu University
13) University of Toyama

14) Nagoya City University
15) Kyoto University

16) Doshisha University

17) Osaka University
18) Osaka Women's and Children's Hospital

19) Hyogo College of Medicine

20) Tottori University
21) Kochi University

22) University of Occupational and Environmental Health, Japan

23) Kyushu University

24) Kumamoto University
25) University of Miyazaki

26) University of the Ryukyus
